# Neuronal Calcium and cAMP Cross-Talk Mediated by Cannabinoid CB_1_ Receptor and EF-Hand Calcium Sensor Interactions

**DOI:** 10.3389/fcell.2018.00067

**Published:** 2018-07-19

**Authors:** Edgar Angelats, Marta Requesens, David Aguinaga, Michael R. Kreutz, Rafael Franco, Gemma Navarro

**Affiliations:** ^1^Molecular Neurobiology Laboratory, Department of Biochemistry and Molecular Biomedicine, University of Barcelona, Barcelona, Spain; ^2^Centro de Investigación en Red, Enfermedades Neurodegenerativas, Instituto de Salud Carlos III, Madrid, Spain; ^3^RG Neuroplasticity, Leibniz Institute for Neurobiology, Magdeburg, Germany; ^4^Leibniz Group “Dendritic Organelles and Synaptic Function”, Center for Molecular Neurobiology Hamburg, University Medical Center Hamburg-Eppendorf, Hamburg, Germany; ^5^Department of Biochemistry and Physiology, Faculty of Pharmacy and Food Science, University of Barcelona, Barcelona, Spain

**Keywords:** caldendrin, calneuron-1, calmodulin, CB_1_ and CB_2_ cannabinoid receptors, development, endocannabinoids, G-protein-coupled receptor, frequenin/NCS1

## Abstract

Endocannabinoids are important players in neural development and function. They act via receptors, whose activation inhibits cAMP production. The aim of the paper was to look for calcium- and cAMP-signaling cross-talk mediated by cannabinoid CB_1_ receptors (CB_1_R) and to assess the relevance of EF-hand CaM-like calcium sensors in this regard. Using a heterologous expression system, we demonstrated that CB_1_R interacts with calneuron-1 and NCS1 but not with caldendrin. Furthermore, interaction motives were identified in both calcium binding proteins and the receptor, and we showed that the first two sensors competed for binding to the receptor in a Ca^2+^-dependent manner. Assays in neuronal primary cultures showed that, CB_1_R-NCS1 complexes predominate at basal Ca^2+^ levels, whereas in the presence of ionomycin, a calcium ionophore, CB_1_R-calneuron-1 complexes were more abundant. Signaling assays following forskolin-induced intracellular cAMP levels showed in mouse striatal neurons that binding of CB_1_R to NCS1 is required for CB_1_R-mediated signaling, while the binding of CB_1_R to calneuron-1 completely blocked G_i_-mediated signaling in response to a selective receptor agonist, arachidonyl-2-chloroethylamide. Calcium levels and interaction with calcium sensors may even lead to apparent Gs coupling after CB_1_R agonist challenge.

## Introduction

The mechanisms involved in the cross-talk at the level of the two main second messengers, Ca^2+^ and cAMP, are not fully elucidated despite being seminal for neuronal function. Regulation of intracellular Ca^2+^ concentration is key for neural development and for brain function and plasticity (Connor et al., 1986; Landfield et al., 1986; Kotlyar and Pivovarov, 1990; Mattson, 1990; Pontzer et al., 1990). Intracellular calcium ion is a second messenger that interacts and affects the structure of a variety of proteins. Although the more studied has been calmodulin (CaM), other calcium binding proteins have been identified and partially characterized (Mikhaylova et al., 2011). Upon ion binding, these calcium-binding proteins are able to participate in a myriad of relevant cellular events, *inter alia* proliferation, differentiation and apoptosis (Hyman and Pfenninger, 1985; Stichel et al., 1987; Forscher, 1989; Joseph et al., 1993; Orrenius and Nicotera, 1994; Orrenius et al., 2003).

Calneuron-1, caldendrin and frequenin are among the calcium-binding proteins abundantly expressed in the central nervous system (CNS). Frequenin, which was identified by its role in the development of the nervous system and the morphology of neurons in snails and fruit flies (see Dason et al., 2012 for review), is also known as neuronal calcium sensor 1 (NCS1). All these proteins share EF-hand domains and regulation of complex neuronal responses that are dependent on calcium gradients. Calneuron-1 presents 4 EF-hand domains, of which only 2 are functional and able to bind calcium with high affinity. Calneuron-1 is a transmembrane protein that binds calcium with considerably higher affinity than NCS1 (Mikhaylova et al., 2006, 2009; McCue et al., 2010; Hradsky et al., 2011; Burgoyne and Haynes, 2012).

Endocannabinoids and *Cannabis sativa*-derived phytocannabinoids act via two G-protein-coupled receptors (GPCRs), CB_1_ and CB_2_ (Matsuda et al., 1990; Griffin et al., 2000; Brown et al., 2002). GPCRs couple to heterotrimeric G proteins, mainly to G_s_, which activates the enzyme that produces the second messenger cAMP, to G_i_, which inhibits the enzyme, or to G_q_ that leads to increases in the level of intracellular calcium ion (Millar and Newton, 2010; Hausch and Holsboer, 2012). CB_1_ is the GPCR with highest expression in CNS neurons and couples to G_i_ proteins. GPCR function may be regulated by interacting proteins (Ritter and Hall, 2009) such as EF-hand CaM like calcium sensors, which may affect the traffick and function of neurotransmitters/neuromodulator GPCRs (Kabbani et al., 2002; Seidenbecher et al., 2004; Mikhaylova et al., 2009; Lian et al., 2011; Navarro et al., 2012, 2014). The aim of this paper was to look for interactions between calcium sensors and the cannabinoid CB_1_ receptor. The results show that endocannabinoid action in neurons is regulated by cAMP-Ca^2+^ cross-talk facilitated by direct receptor/calcium sensor protein interactions.

## Materials and methods

### Reagents

Ionomycin calcium salt from *Streptomyces conglobatus* was purchased from SigmaAldrich (St Louis, MO), and Arachidonyl-2′-chloroethylamide (ACEA) and rimonabant from Tocris Bioscience (Bristol, UK).

### Expression vectors

Plasmids containing the human version of all proteins, coding for either full-length or point or deletion mutants were used. In the case of the human CB_1_R (obtained from cDNA resource center), two mutants were used. One of them (CB1^IL3^) had mutations in the residues susceptible of phosphorylation located in the third intracellular loop (from ^321^TSEDGKVQV^329^ to ^321^AAEDGKVQV^329^); they were prepared as described elsewhere by site-directed mutagenesis (Cellogenetics, Ijamsville, MD, USA; see Navarro et al., 2010). PCR was used to remove the last 40 amino acids of the CB_1_R (${\text{CB}}_{1}^{\Delta C}$). cDNA for the human version of cannabinoid CB_1_R (mutated or not) without its stop codon, were subcloned to RLuc-containing vector (p*RLuc*-N1; PerkinElmer, Wellesley, MA) using sense and antisense primers harboring unique restriction sites for HindIII and BamHI or subcloned to pEYFP-containing vector (pEYFP-N1; Clontech, Heidelberg, Germany) using sense and antisense primers harboring unique restriction sites for BamHI and KpnI generating CB_1_R-RLuc and CB_1_R-YFP. cDNA constructs encoding NCS1 or caldendrin in pcDNA3 vectors, kindly provided by Prof. Michael R. Kreutz were subcloned in pEYFP-N1 vector as previously described (Navarro et al., 2012) to generate NCS1-YFP and caldendrin-YFP, fusion proteins. Calneuron-1 sequence, cloned into pcDNA3.1 (provided by Prof. Kreutz), was amplified without its stop codon using sense and antisense primers harboring unique BamHI and HindIII sites to subclone calneuron-1 in p*RLuc*-N1vector or HindIII and BamHI to subclone calneuron-1 in pEYFP-N1 vector, giving the plasmids that express calneuron-1-RLuc and calneuron-1-YFP. Calneuron-1 construct truncated in the C terminus (calneuron-1^Δ*C*^) and N-terminal myristoylation-deficient NCS1 mutant (NCS1^Δ*myristoil*^) in which YFP is fused to the N terminus of NCS1 obtained as described elsewhere (Hradsky et al., 2013) were kindly provided by Michael R. Kreutz. Fusion proteins corresponding to mutant sensors were obtained following the above described methodology.

### Cell culture and transient transfection

HEK-293T cells were grown in DMEM medium (Gibco, Paisley, Scotland, UK) supplemented with 2 mM L-glutamine, 100 U/ml penicillin/streptomycin, MEM non-essential amino acids solution (1/100) and 5% (v/v) heat inactivated fetal bovine serum (FBS) (Invitrogen, Paisley, Scotland, UK). Cells were maintained in a humid atmosphere of 5% CO_2_ at 37°C. Cells were transiently transfected with the PEI (PolyEthylenImine, Sigma, St. Louis, MO, USA) method as previously described (Navarro et al., 2015). The efficiency of PEI-based transfection was >60% (even in co-transfected cells). Primary cultures of striatal neurons were obtained from fetal CD-1 mice (embryonic day 16; E16) (Penrod et al., 2011). In brief, after removing brain meninges, striatal tissue was dissected and digested in 0.25% trypsin for 15 min. Trypsinization process was stopped by aspirating the media and washing three times with filtrated HBSS solution. After passage through a 100 μm-pore mesh and pelleting (3 min at 300 x g), neurons were resuspended in DMEM medium and seeded in poly-D-lysine coated 6-well plates at 500.000 cells/well or 100.000 cells/18 mm coverslip. 24 h later, the medium was replaced and cells were maintained in Neurobasal medium (Gibco; Paisley, Scotland, UK) supplemented with 5% FBS, 2 mM L-glutamine, 100 U/ml P/S and 2% (v/v) B27 supplement. The cells were maintained at 37°C in humidified 5% CO_2_ atmosphere for 12 days before utilization, being the medium replaced once a week.

### Bioluminescence resonance energy transfer (BRET) assays

HEK-293T cells were transiently cotransfected with a constant amount of cDNA encoding for either CB_1_-RLuc, calneuron-1-RLuc or NCS1-RLuc and increasing amounts of cDNAs for caldendrin-YFP, NCS1-YFP, calneuron-1-YFP, NCS1^Δmyristoil^-YFP, calneuron-1^ΔCT^-YFP, ${\text{CB}}_{1}^{\Delta C}$-YFP or ${\text{CB}}_{1}^{\text{IL}3}$-YFP. 48 h after transfection cells were adjusted to 20 μg of protein using a Bradford assay kit (Bio-Rad, Munich, Germany) and using bovine serum albumin for standardization. To quantify protein-YFP expression, fluorescence was read in a Mithras LB 940 (Berthold Technologies, DLReady, Germany). For BRET measurements, readings were collected 1 min after the addition of 5 μM coelenterazine H (P.J.K. GmbH; Kleinblittersdorf, Germany) using a Mithras LB 940, which allows the integration of the signals detected in the short-wavelength filter at 485 nm and the long-wavelength filter at 530 nm. To quantify protein-RLuc expression, luminescence readings were performed 10 min after coelenterazine H addition. The net BRET is defined as [(long-wavelength emission)/(short-wavelength emission)]-C_f_, where C_f_ corresponds to [(long-wavelength emission)/(short-wavelength emission)] for the donor construct expressed alone in the same experiment. GraphPad Prism software (San Diego, CA, USA) was used to fit data. BRET is expressed as milli BRET units, mBU (net BRET × 1,000).

### Western blotting

The determination of protein expression levels by immunoblotting was carried out in transiently transfected HEK-293T cells. 48 h after transfection, cells were collected, centrifuged and resuspended in ice-cold Tris-HCl buffer pH 7.4 containing protease inhibitor (1/1,000). Then, cell suspension was disrupted with a Polytron homogenizer (PTA 20 TS rotor, setting 3; Kinematica, Basel, Switzerland) for three 5-s periods, and membranes were obtained by centrifugation at 16,000 g (30 min, 4°C). Pellet was resuspended in PBS-NP-40 1% buffer for 1h. After centrifugation at 16,000 g (30 min, 4°C), protein was quantified by the bicinchoninic acid method (Pierce Chemical Co., Rockford, IL, USA) using BSA dilutions as standard. Equivalent amounts of protein (20 μg) were separated by polyacrylamide gel electrophoresis on denaturating conditions (10% SDS). Proteins in gels were transferred into PVDF membranes, which were then treated with blocking solution and PBS (1:1 v/v) for 1 h. Primary antibodies against NCS1 (rabbit anti NCS1, 1/1,200; AbCam) or against calneuron-1 (rabbit anti calneuron-1, 1/2,000; AbCam) diluted in PBS buffer were added and kept over-night at 4°C. After removal of the primary antibody and several washes with PBS-Tween 20 0.05%, membranes were incubated for 1 h with a goat-anti-rabbit IRDye 680RD secondary antibody (LI-COR Bioscience, Lincoln, USA; dilution 1:10,000). Several washes were performed before quantification using an Odyssey infrared scanner (LI-COR Bioscience, Lincoln, USA) and the Odyssey software.

### Immunocytochemistry

Transfected HEK-293T cells were fixed in 4% paraformaldehyde for 15 min and washed three times with PBS containing 20 mM glycine before permeabilization with PBS-glycine containing 0.2% Triton X-100 (10 min incubation). Cells were treated for 1 h with PBS containing 1% bovine serum albumin and labeled overnight with the mouse anti-RLuc primary antibody (1/50; Millipore), and subsequently treated with a Cy3-conjugated anti-mouse secondary antibody [1/200; Jackson ImmunoResearch (red)] IgG (1 h each). Samples were washed several times and mounted with 30% Mowiol (Calbiochem). Samples were observed in a Leica SP2 confocal microscope (Leica Microsystems). YFP-fused protein were detected by the yellow fluorescence. Nuclei were stained with Hoechst (SigmaAldrich, 1/100). Scale bar: 20 μm.

### cAMP determination

Two hours before initiating the experiment, neuron culture medium was replaced by serum-starved DMEM medium. Then, cells were detached and resuspended in serum-starved DMEM medium containing 50 μM zardaverine, 0.1% BSA and 5mM HEPES. Cells were plated in 384-well microplates (2,000 cells/well), pretreated (15 min) with the corresponding antagonists -or vehicle- and stimulated with agonists (15 min) before adding 0.5 μM forskolin (15 min). Readings were performed after 1 h incubation at 25°C. Homogeneous time-resolved fluorescence energy transfer (HTRF) measures were performed using the Lance Ultra cAMP kit (PerkinElmer, Waltham, MA, USA). According to the manufacturer and to our previous experience phenol red in DMEM does not affect cAMP concentration determinations. Fluorescence at 665 nm was analyzed on a PHERAstar Flagship microplate reader equipped with an HTRF optical module (BMG Lab technologies, Offenburg, Germany).

### *In situ* proximity ligation assay (PLA)

Neurons grown on glass coverslips were fixed in 4% paraformaldehyde for 15 min, washed twice with PBS containing 20 mM glycine to quench the aldehyde groups and permeabilized with the same buffer containing 0.05% Triton X-100 (10 min treatment). After 1 h incubation at 37° with blocking solution, cells were treated with specific antibodies against CB_1_ (rabbit anti CB_1_R, 1/100; Invitrogen), calneuron-1 (rabbit anti calneuron-1, 1/100; AbCam) and NCS1 (rabbit anti NCS1, 1/100; AbCam) conjugated with Duolink *In Situ* Probemaker oligonucleotides (CB_1_R was labeled with Duolink *In Situ* Probemaker Minus and anti-NCS1 and anti-calneuron-1 antibodies were labeled with Duolink *In Situ* Probemaker Plus). Nuclei were stained with Hoechst (1/200; SigmaAldrich) and samples were mounted with 30% Mowiol (Calbiochem) and observed in a Leica SP2 confocal microscope (Leica Microsystems, Mannheim, Germany) equipped with an apochromatic 63X oil-immersion objective using 405 nm and 594 nm laser lines.

For each field of view a stack of two channels (one per staining) and four stacks with a step size of 0.5 μm were acquired. The number of cells containing one or more red spots vs. total cells (blue nucleus) and, in cells containing spots, the ratio r (number of red spots/cell), were determined by means of the Duolink Image tool software.

## Results

### EF-hand CaM-like sensors interact with the CB_1_R

**Figure 1:**
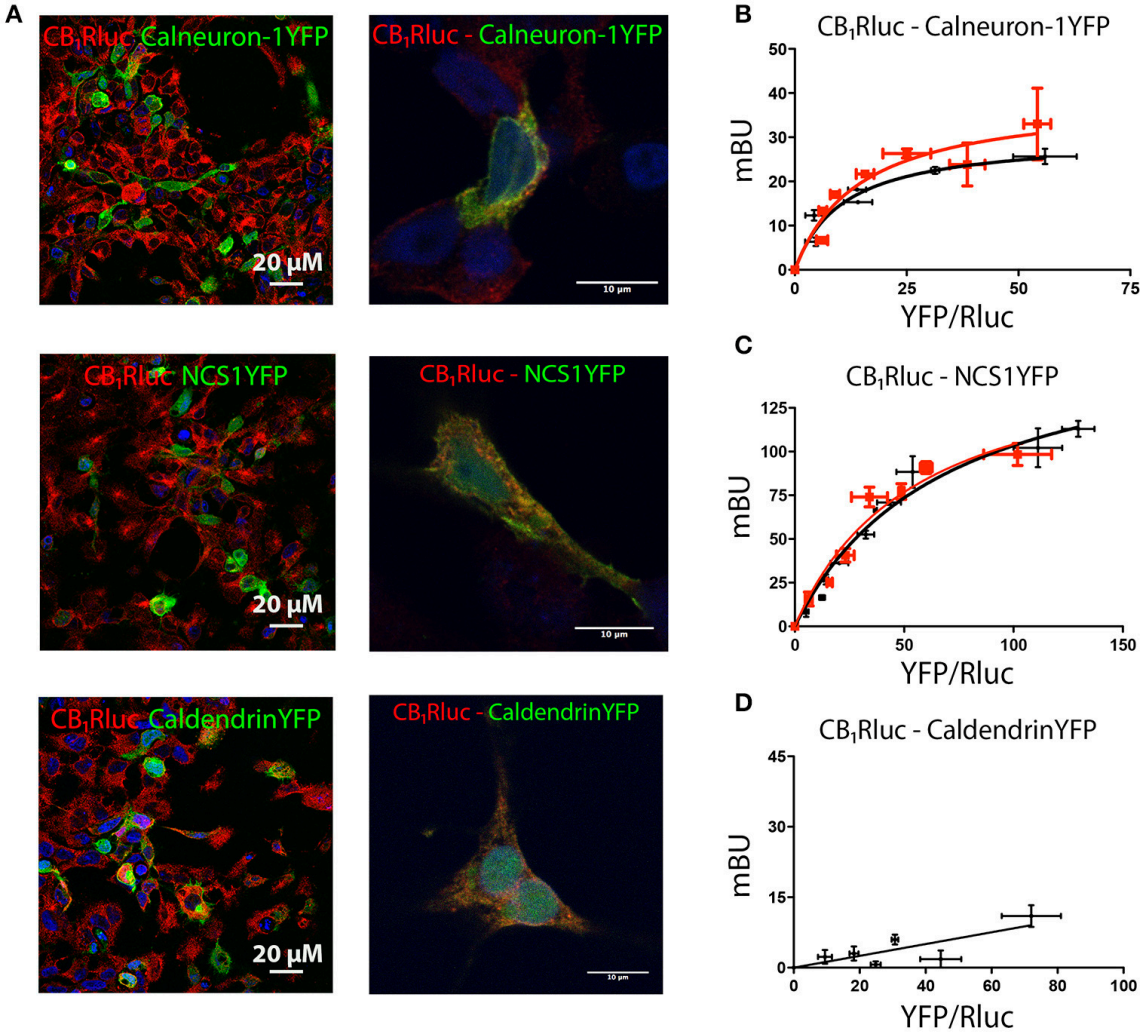
Interaction of CB_1_R with calcium sensor proteins. For immunocytochemistry assays HEK-293T cells were co-transfected with cDNAs for CB_1_-RLuc (1.5 μg) and calneuron-1-YFP (2 μg), CB_1_-RLuc (1.5 μg) and NCS1-YFP (2 μg) or CB_1_-RLuc (1.5 μg) and caldendrin-YFP (3 μg). Cells were processed as described in section Materials and Methods. **(A)** CB_1_-RLuc was detected using a specific anti-RLuc antibody and a Cy3-conjugated secondary antibody, whereas sensor-YFP fusion proteins were detected by the yellow fluorescence due to YFP; merge is shown in images in the right, in which colocalization appears in yellow. Lower magnification (left) and high magnification images (right) are shown. **(B–D)** BRET assays were performed in HEK-293T cells transfected with cDNA for CB_1_-RLuc (1.5 μg) and increasing amounts of cDNAs for calneuron-1-YFP (0.25–2 μg) **(B)**, NCS1-YFP (0.5–2.5 μg) **(C)** or caldendrin-YFP (1–3 μg) **(D)**, in the presence (red) or absence (black) of 1 μM ionomycin. Values are the mean ± S.E.M. of 10 different experiments in duplicates.

To determine whether CB_1_R can form heteromeric complexes with calcium-binding proteins, an immunocytochemistry assay was first developed to assess whether CB_1_R and three calcium sensors of relevance in neurons, may co-localize in co-transfected cells. HEK-293T cells were transfected with cDNAs for CB_1_-RLuc, and either calneuron-1-YFP, NCS1-YFP or caldendrin-YFP. Calcium-binding protein expression was detected by fluorescence while CB_1_R fused to RLuc was detected by the use of an anti-RLuc antibody and a Cy3-conjugated secondary antibody. Figure 1A shows expression of proteins in cells transfected with cDNAs encoding for CB_1_-RLuc and a calcium-sensor fused to YFP. Cell expression was similar to that observed when cells were transfected with only one fusion protein (Supplementary Figure 1). The degree of overlapping of immunoreactivity was moderate for CB_1_ and each of the calcium sensors tested (Figure 1A). To demonstrate a potential physical interaction between receptor pairs, a bioluminescence resonance energy transfer (BRET) approach was used. BRET was undertaken in HEK-293T cells expressing a constant amount of cDNA for CB_1_-RLuc and increasing amounts of cDNAs for calneuron-1-YFP, NCS1-YFP or caldendrin-YFP. For calneuron-1-YFP and NCS1-YFP a saturation BRET curve was obtained, thus indicating a specific interaction between CB_1_-RLuc and those two calcium binding proteins. BRET parameter values were: BRET_max_ 30 ± 3 mBU and BRET_50_ 10± 3 for the interaction with calneuron-1, and BRET_max_ 170 ± 20 mBU and BRET_50_ 70 ± 10 for the interaction with NCS1 (Figures 1B,C). Interestingly, the increase of intracellular Ca^2+^ concentration by pre-incubation for 30 min with ionomycin, a ionophore that increases intracellular calcium ion levels, did not modify the degree of interaction (Figures 1B,C). In contrast, an unspecific linear signal was obtained between CB_1_ and caldendrin-YFP, indicating the lack of interaction between these proteins (Figure 1D). Actually, these results constitute a proper negative control of the assay. In summary, the results indicate that at basal or increased Ca^2+^ intracellular levels, CB_1_R may interact with calneuron-1 or NCS1 but not with caldendrin.

### Mapping the interaction motives

**Figure 2:**
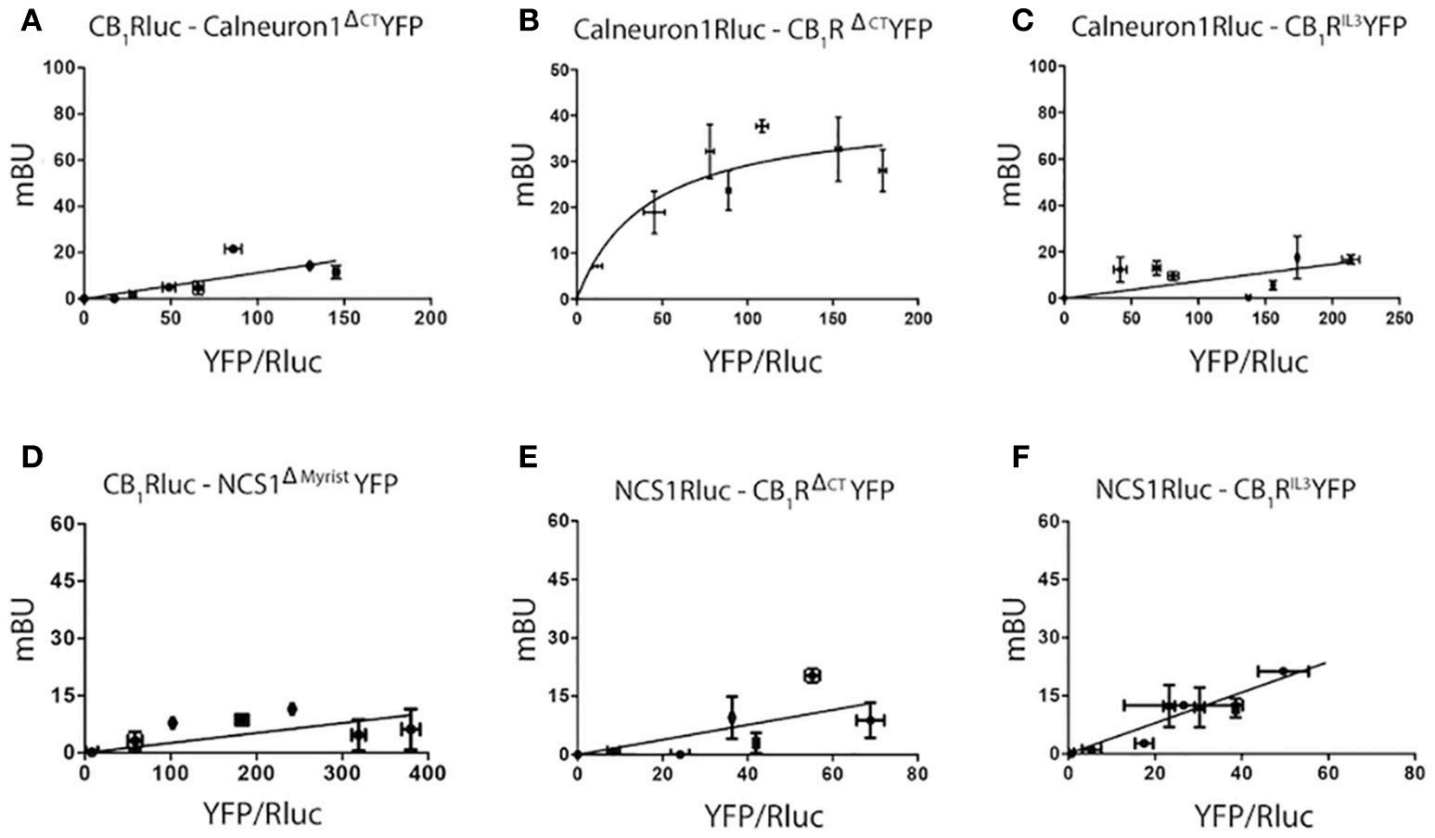
Determination of interacting domains for CB_1_R-calneuron-1 and CB_1_R-NCS1 complexes. BRET assays were performed in HEK-293T cells transfected with cDNA for CB_1_-RLuc (1.5 μg) and increasing amounts of cDNA for calneuron-1^ΔCT^YFP (1–3 μg) **(A)**, cDNA for calneuron-1RLuc (3 μg) and increasing amounts of cDNA for ${\text{CB}}_{1}R^{\Delta CT}$YFP (0.25–1.5 μg) **(B)**, cDNA for calneuron-1-RLuc (3 μg) and increasing amounts of cDNA for ${\text{CB}}_{1}{\text{R}}^{\text{IL}3}$YFP (0.5–2 μg) **(C)**, cDNA for CB_1_-RLuc (1.5 μg) and increasing amounts of cDNA for NCS1^Δmyristoil^YFP (1–3 μg) **(D)**; cDNA for NCS1-RLuc (2.5 μg) and increasing amounts of cDNA for CB_1_R^ΔCT^YFP (0.25–1.5 μg) **(E)** and cDNA for NCS1-RLuc (2.5 μg) and increasing amounts of cDNA for CB_1_R^IL3^YFP (0.5–2 μg) **(F)**. Values are the mean ± S.E.M. of 8 different experiments in duplicates.

Taking into account data on motives in the structure of calcium sensors and the general organization of heptaspanning GPCRs, BRET assays were undertaken using different mutated versions of CB_1_R and calneuron-1 or NCS1. As indicated in Material and Methods, plasmids containing the sequences of either the third intracellular loop or the C-terminal deletion mutants of CB_1_R were used (see details in section Methods). For calcium sensors, NCS1 was mutated deleting the myristoylation site in the N-terminal domain of NCS1, and a part of the C-terminal end of calneuron-1 was deleted to prevent membrane insertion. Images showing expression of mutated proteins are displayed in Supplementary Figure 2. BRET assays using RLuc or YFP fusion proteins containing the mutated proteins led to the results displayed in Figure 2. On the one hand, the BRET results show that mutant versions of calneuron-1 and NCS1 cannot interact with the CB_1_R. On the other hand, the mutated form in the third intracellular loop of the CB_1_R receptor abolished the interaction with both calcium sensor proteins. Finally, deletion of the C-terminal domain led to unspecific signal when NCS1-YFP was used but did not significantly alter the direct interaction established between calneuron-1-YFP and the cannabinoid receptor. These results suggest that NCS1 directly interact with the C-terminal tail and the third intracellular loop of the CB_1_R, while calneuron-1 only interacts with the C-terminal domain of the receptor.

### Calneuron-1 and NCS1 compete for the interaction with CB_1_R

**Figure 3:**
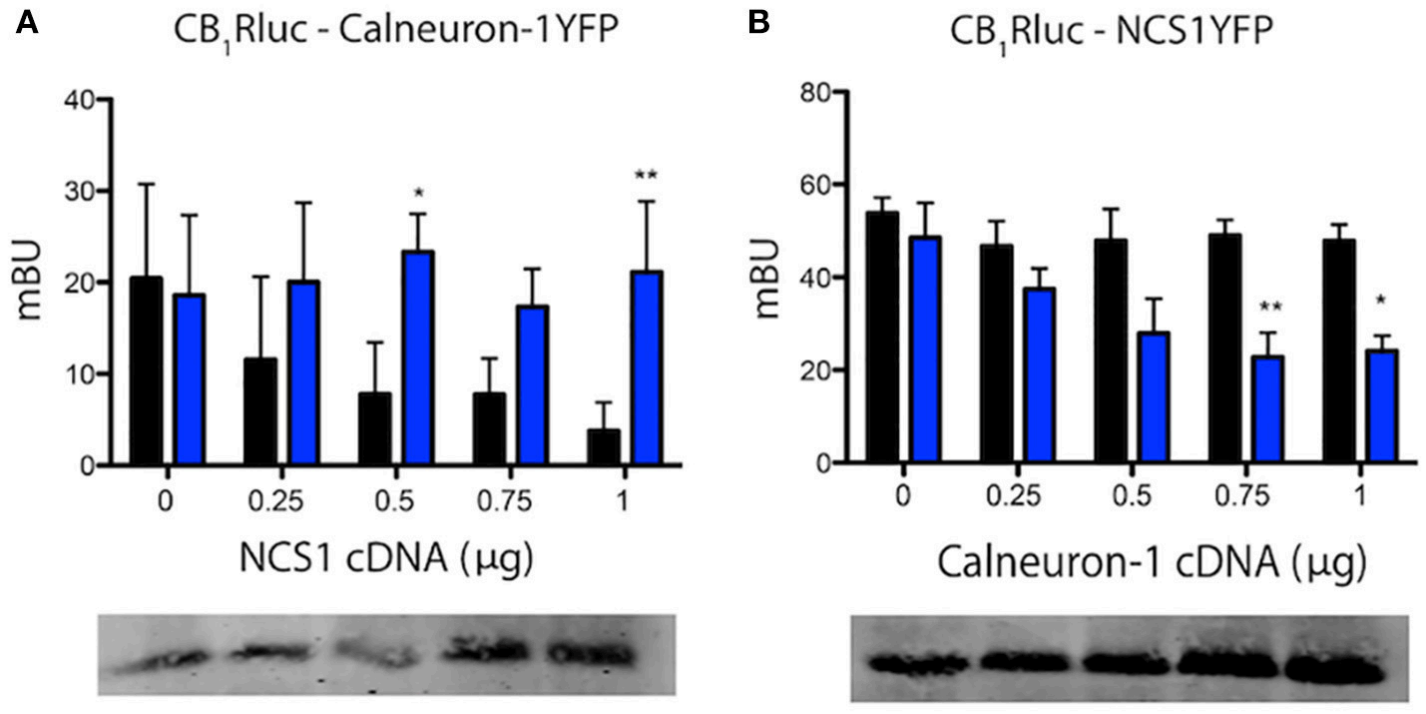
Calneuron-1 and NCS1 compete for interacting with CB_1_R. BRET assays were performed in HEK-293T cells transfected with cDNAs for CB_1_R (1.25 μg) and calneuron-1-YFP (2 μg) and increasing amounts of cDNAs for NCS1 (0–1 μg) **(A)**, for CB_1_R (1.25 μg) and NCS1-YFP (1.5 μg) and increasing amounts of cDNA for calneuron-1 (0–1 μg) **(B)**, in the presence (blue) or absence (black) of 1 μM ionomycin. Bottom images show the results of a representative Western blotting in which NCS1 or calneuron-1 were detected. Values are the mean ± S.E.M. of 10 different experiments in duplicates and a one-way ANOVA followed by a Dunnett's *post-hoc* test was used for statistical comparison (*p < 0.05; **p < 0.01 comparing absence vs. presence of ionomycin).

The above described results concerning G-protein-mediated signaling suggested that the two calcium-binding proteins might compete for binding to the cannabinoid receptor. To confirm this hypothesis, we performed BRET competition assays by expressing CB_1_-RLuc and one sensor fused to YFP in the presence of increasing amounts of the second calcium sensor. BRET assays were performed in cells treated or not with ionomycin and the results are presented in Figure 3. Results using increasing amounts of cDNA for NCS1 indicate that the protein was able to dose-dependently compete for the binding of calneuron-1 to CB_1_R; such competence disappeared in the presence of increased intracellular Ca^2+^ concentrations (Figure 3A). Just the opposite was found when using increasing amounts of cDNA for calneuron-1, which did compete for the binding of NCS1 to the receptor when the concentrations of intracellular Ca^2+^ were elevated (Figure 3B). The results indicate that in basal conditions CB_1_R is more likely interacting with NCS1, while relatively high cytoplasmic calcium ion concentrations favor the binding of calneuron-1 to the receptor.

### Occurrence of receptor-calcium sensor complexes in striatal neurons

**Figure 4:**
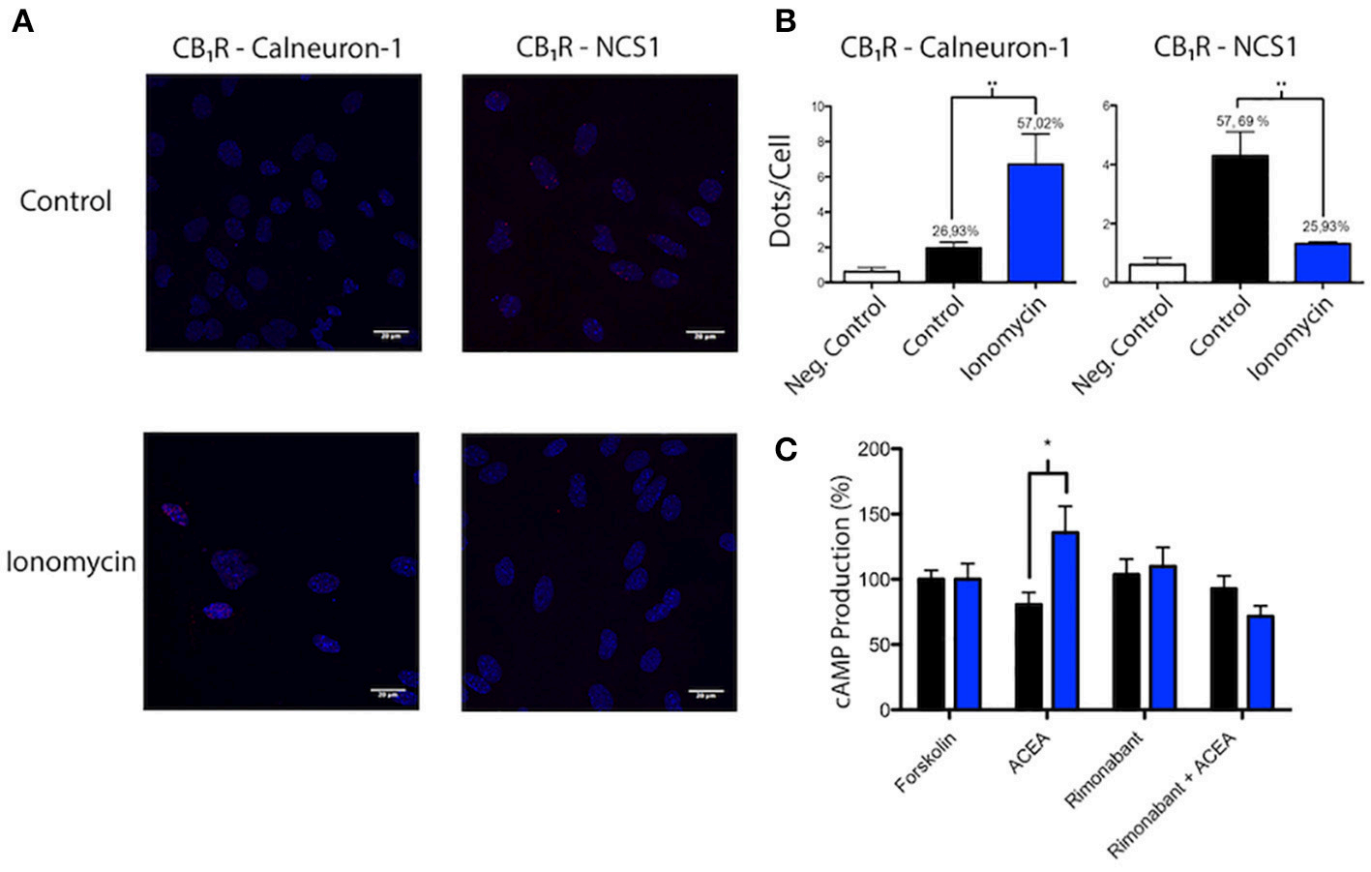
Identification of interactions between calcium sensor proteins and CB_1_R in primary cultures of mouse striatal neurons. **(A,B)** *In situ* PLA detection of CB_1_R-calneuron-1 and CB_1_R-NCS1 complexes was performed as described in Material and Methods using primary cultures assayed in the presence or absence of 1 μM ionomycin. Interactions were detected as red spots in Hoechst-stained nuclei in the confocal microscopy images shown (superimposed sections of 0.5 μm total thickness). Scale bar: 20 μm **(A)**. Dots (number of red spots) per cell and percentage of cells containing red spots are shown in the bar graphs. Data are the mean ± S.E.M of counts of 8 different fields for every condition. A one-way ANOVA followed by a Tukey's multiple comparison test was performed for the statistical analysis (*p < 0.05; **p < 0.01) **(B)**. **(C)** Effect of 100 nM ACEA on 0.5 μM forskolin-induced intracellular cAMP levels determined in striatal neurons pretreated or not with 1 μM rimonabant. Basal and forskolin-induced concentrations were, respectively 0.3 ± 0.2 and 3.7 ± 0.5 nM. Experiments were carried out in the absence (black) and presence (blue) of a calcium ionophore (1 μM ionomycin). Data are given as percentage of cAMP concentration induced by forskolin; data are the mean ± S.E.M. of 6 different experiments in triplicates. A one-way ANOVA followed by a Dunnett's *post-hoc* test was used for statistical comparison (*p < 0.05 comparing absence vs. presence of ionomycin).

Mouse striatum was chosen to prepare neuronal primary cultures and look for interactions between the CB_1_R and calneuron-1 or NCS1. In order to detect receptor-sensor complexes in primary cultures of neurons, PLA assays were carried out in cells treated or not with ionomycin. Red dots/clusters indicating the occurrence of receptor-calneuron-1 and receptor-NCS1 complexes were found in all conditions (Figure 4A). The absence of the primary anti-CB_1_R or anti-sensor antibodies led to a marked reduction of the PLA signal (Figure 4B, Supplementary Figure 3). The number of cells containing one or more red spots vs. total cells (blue nucleus) and, in cells containing spots, the ratio r (number of red spots/cell), were determined by means of the Duolink Image tool software. Significant differences were found in both basal conditions and after treatment with ionomycin. In basal conditions, the number of cells expressing CB_1_R-calneuron-1 complexes and the relative number of clusters was lower than in the case of the CB_1_R-NCS1 pair. The results were totally opposite when neurons were treated with ionomycin, which led to a marked reduction in the percentage of cells expressing CB_1_R/NCS1 complexes (and in the spot/cell ratio) and a marked increase in the percentage of cells expressing CB_1_R/calneuron-1 complexes (and in the spot/cell ratio) (Figure 4B). Collectively, the results indicate that low calcium concentrations favor the interaction with NCS1, whereas CB_1_R is mainly bound to calneuron-1 when intracellular Ca^2+^ levels increase (Figure 4B). It should be noted that the effect of ACEA, the CB_1_R agonist, was markedly affected by preincubation of neurons with ionomycin (Figure 4C). Of note, the combination of calcium mobilization and receptor activation results in higher cAMP levels being the net effect equivalent to a G_s_ coupling and not to a G_i_ coupling as it occurred in basal conditions.

## Discussion

GPCR receptor action may be modulated in different ways depending on the cellular context. GPCR-mediated signaling not only depends on the coupled G protein but on other molecules able to interact with the receptor and/or with the signaling machinery. The potential relationships between calcium-binding proteins and GPCRs are poorly understood despite pioneering articles showing that calmodulin is involved in hormone action (Means and Dedman, 1980), regulates GPCR kinases (Iacovelli et al., 1999) and impacts on the activity of enzymes that regulate cAMP levels after activation of dopamine and opioid receptors (Hanbauer et al., 1979). Calmodulin was likely the first calcium-binding protein that was identified as a modulator of GPCRs relevant for neurotransmission (Woods et al., 2008; Navarro et al., 2009; Ferré et al., 2010; Fuxe et al., 2010; Chen et al., 2011; Magalhaes et al., 2012). However, the knowledge about the impact in GPCR biology of other neuronal calcium sensors is still preliminary (Mikhaylova et al., 2011). In this study, we aimed at looking for interactions between the most abundant GPCR in the CNS, CB_1_R and three abundantly expressed calcium sensors (NCS1, calneuron-1 and caldendrin) with known function. Caldendrin is highly enriched at synaptic sites (Seidenbecher et al., 1998) and binds various synaptic proteins (Seidenbecher et al., 2004; Dieterich et al., 2008; Gorny et al., 2012; Mikhaylova et al., 2018). The results showed interaction of the GPCR with calneuron-1 and NCS1 but not with caldendrin, and competence of calneuron-1 and NCS1 for the binding to the receptor that was calcium-dependent. To our knowledge no interaction between caldendrin and any GPCR has been reported.

It has been previously described that NCS1 can interact with dopamine D_2_ (Woll et al., 2011) and adenosine A_2A_ (Navarro et al., 2012) receptors. In turn, calneuron-1 can also establish interactions with the A_2A_−*D*_2_ receptor heteromer (Navarro et al., 2014). The cannabinoid CB_1_R is involved in different interaction whose interface motives are partially characterized. Intracellular loops (particularly the 2nd and the 3rd) are involved in coupling to heterotrimeric G proteins and also to other scaffolding and/or signaling proteins (Khan and Lee, 2014). Also, the C-terminal domain of GPCRs arise as important for directing signaling to different pathways thus contributing to functional selectivity (Stadel et al., 2011; Navarro et al., 2018). It has been also suggested that structural features in the 2nd intracellular loop may be responsible for a change of coupling from the cognate G_i_ to a G_s_ protein (Chen et al., 2010) and that the third intracellular loop is important for constitutive activation (Abadji et al., 2008). Based on preliminary assays and on previous data we tested CB_1_ receptors that were mutated in amino acids that are responsible for electrostatic interaction with other proteins (Navarro et al., 2010). Plasmids were prepared containing the CB_1_R in which two conserved residues that are susceptible of phosphorylation (thus acquiring negative charges) were mutated to Ala. Similar mutations were done in peptide strings in both the third intracellular loop (${\text{CB}}_{1}R^{\text{IL}3}$) and the C-terminal end (${\text{CB}}_{1}R^{\Delta C}$). One of the remarkable findings was the asymmetric interaction mode with calcium sensors; whereas mutations in the third intracellular loop abolished binding to the two calcium-binding proteins, mutations in the C-terminal domain abolished interaction with NCS1 but not with calneuron-1. In summary, we were able to achieve one of our interests, which was to look for structural differences in the interaction between the CB_1_R and calneuron-1 or NCS1.

We here demonstrate that CB_1_R function might be regulated by direct interactions with calcium sensors that in turn depend on the level of intracellular Ca^2+^. It is also demonstrated that the Gi- coupled CB_1_R is functionally affected by the calcium concentration and by the interaction with calcium sensors in such a way that the output may be similar to that of G_s_-coupled receptors. Calcium sensors may also modulate G_s_-coupled GPCRs. For instance, in the case of G_s_-coupled adenosine A_2A_ receptors, NCS1 is the protein that modulates receptor signaling in a calcium concentration-dependent fashion (Navarro et al., 2012). Taken together, the evidence points to Ca^2+^ levels as relevant to regulate the function of receptors whose signaling is dependent on cAMP as second messenger.

## Author contributions

RF and GN designed and co-directed the research and wrote the manuscript. MK provided reagents, provided knowledge on calcium-binding protein cell, structural and molecular biology, and contributed to manuscript editing and interpretation of data. MR and EA participated in the performance of experiments and were helped by DA in all assays involving primary cultures of neurons. MR, EA, and DA wrote the Methods section and contributed to manuscript editing.

### Conflict of interest statement

The authors declare that the research was conducted in the absence of any commercial or financial relationships that could be construed as a potential conflict of interest.

## Supplementary material

Supplementary Figure 1 Expression of CB_1_R and calcium sensor fusion proteins. HEK-293T cells were transfected with cDNAs for CB_1_-RLuc (1 μg) **(A,E**,**F)**, calneuron-1-YFP (1.5 μg) **(B)**, NCS1-YFP (1.5 μg) **(C)** or caldendrin-YFP (1.75 μg) **(D)**. Immunocytochemical assays were performed as described in section Materials and Methods. In order to ensure the specificity of the antibodies employed, negative controls were developed by omitting the primary anti-RLuc antibody **(E)** or the Cy3-conjugated secondary antibody **(F)**. Scale bar: 30 μm.

Supplementary Figure 2 Expression of calcium sensor fusion protein mutants. HEK-293T cells were transfected with plasmids containing, for CB_1_R, the sequence containing mutations forms in the third intracellular loop and the sequence lacking the C-terminal domain; for NCS1, the sequence lacking the myristoylation site in the N-terminal domain of NCS1 and, for calneuron-1, the sequence lacking of the C-terminal domain involved in membrane insertion. Detection was performed by fluorescence using a confocal microscope. Scale bars: 10 μm.

Supplementary Figure 3 Negative controls in PLA Assays. Negative controls for *in situ* PLA assays were performed in primary cultures of mice striatal neurons by omitting either the anti-CB_1_R **(A)**, the anti-NCS1 **(B)** or the anti-calneuron-1 **(C)** antibodies. Scale bar: 30 μm.
