# Supplementary figures and images for: Neuronal Calcium and cAMP Cross-Talk Mediated by Cannabinoid CB1 Receptor and EF-Hand Calcium Sensor Interactions
Source: Front Cell Dev Biol. 2018 Jul 19;6:67. doi: 10.3389/fcell.2018.00067 (PMC6060245; doi:10.3389/fcell.2018.00067)

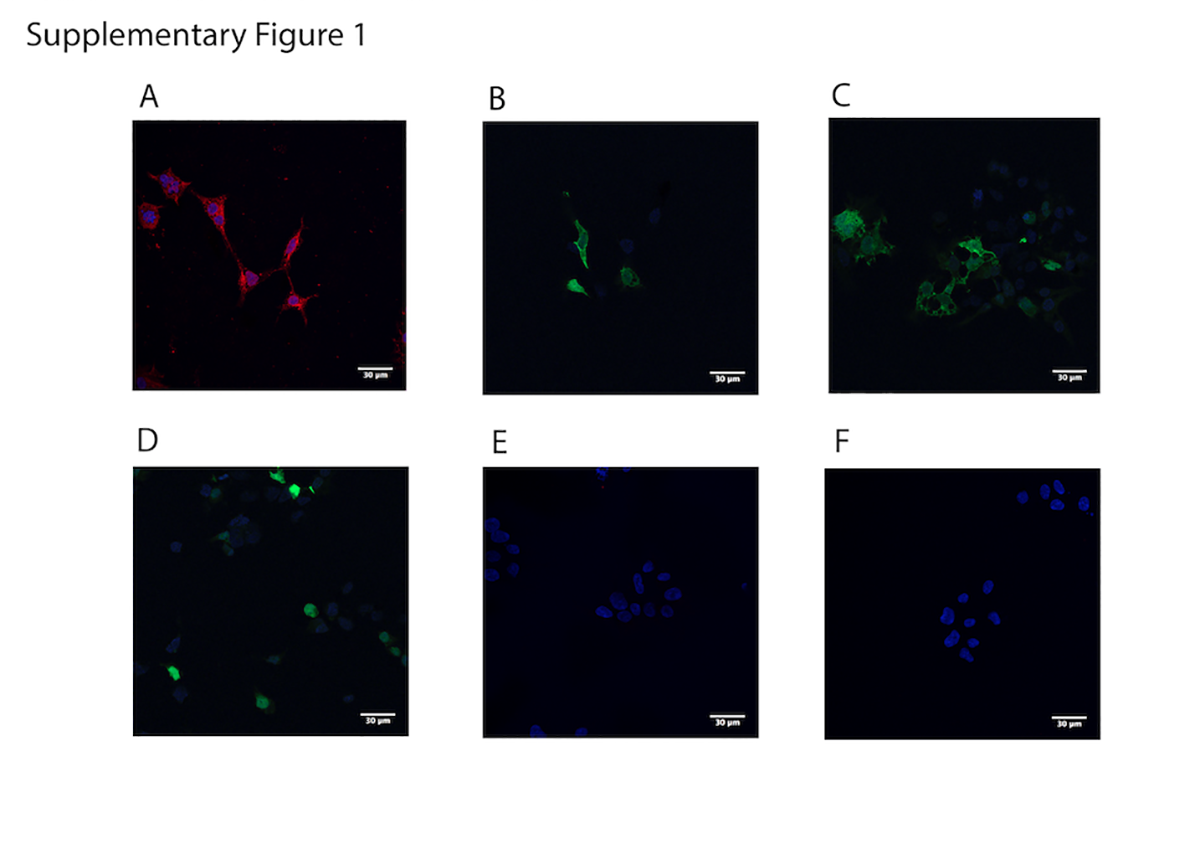

Supplement: Supplementary Figure 1 — Expression of CB1R and calcium sensor fusion proteins. HEK-293T cells were transfected with cDNAs for CB1-RLuc (1 μg) (A,E,F), calneuron-1-YFP (1.5 μg) (B), NCS1-YFP (1.5 μg) (C) or caldendrin-YFP (1.75 μg) (D). Immunocytochemical assays were performed as described in section Materials and Methods. In order to ensure the specificity of the antibodies employed, negative controls were developed by omitting the primary anti-RLuc antibody (E) or the Cy3-conjugated secondary antibody (F). Scale bar: 30 μm. [file Image_1.TIFF]

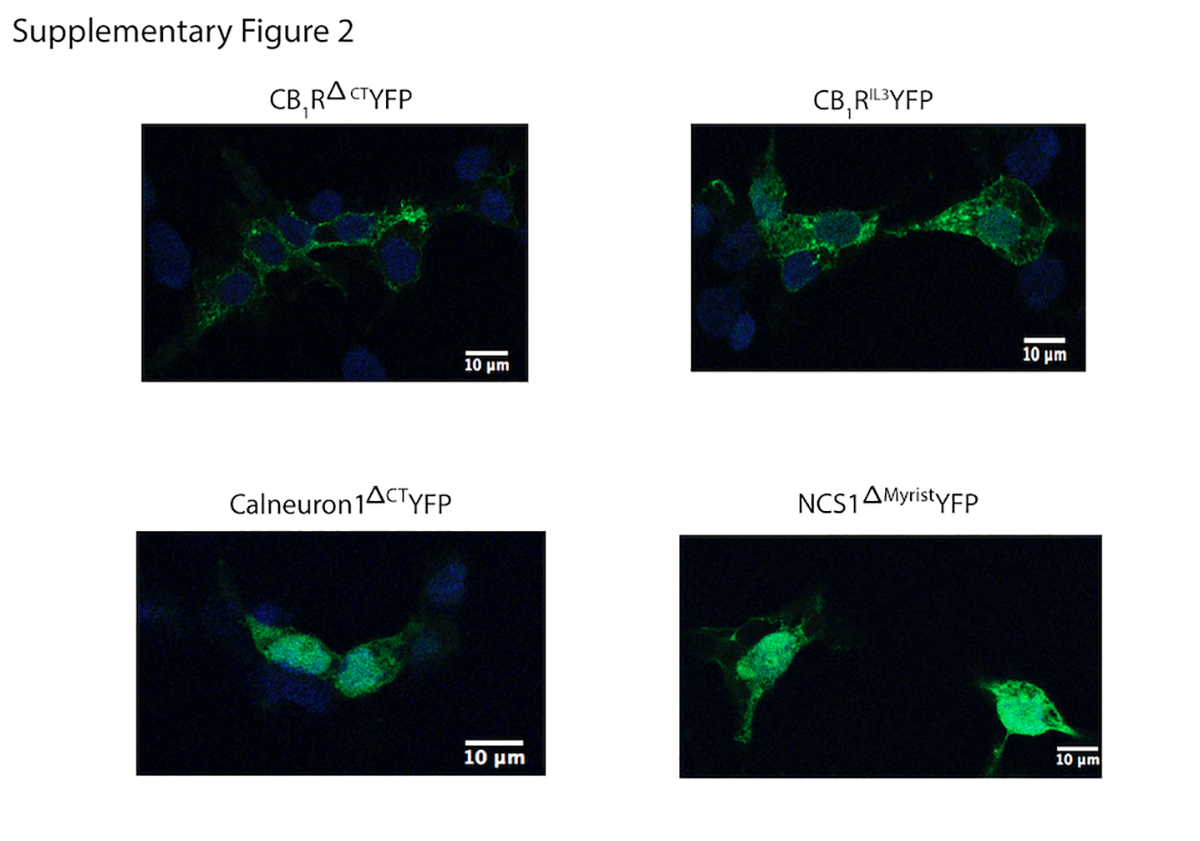

Supplement: Supplementary Figure 2 — Expression of calcium sensor fusion protein mutants. HEK-293T cells were transfected with plasmids containing, for CB1R, the sequence containing mutations forms in the third intracellular loop and the sequence lacking the C-terminal domain; for NCS1, the sequence lacking the myristoylation site in the N-terminal domain of NCS1 and, for calneuron-1, the sequence lacking of the C-terminal domain involved in membrane insertion. Detection was performed by fluorescence using a confocal microscope. Scale bars: 10 μm. [file Image_2.TIF]

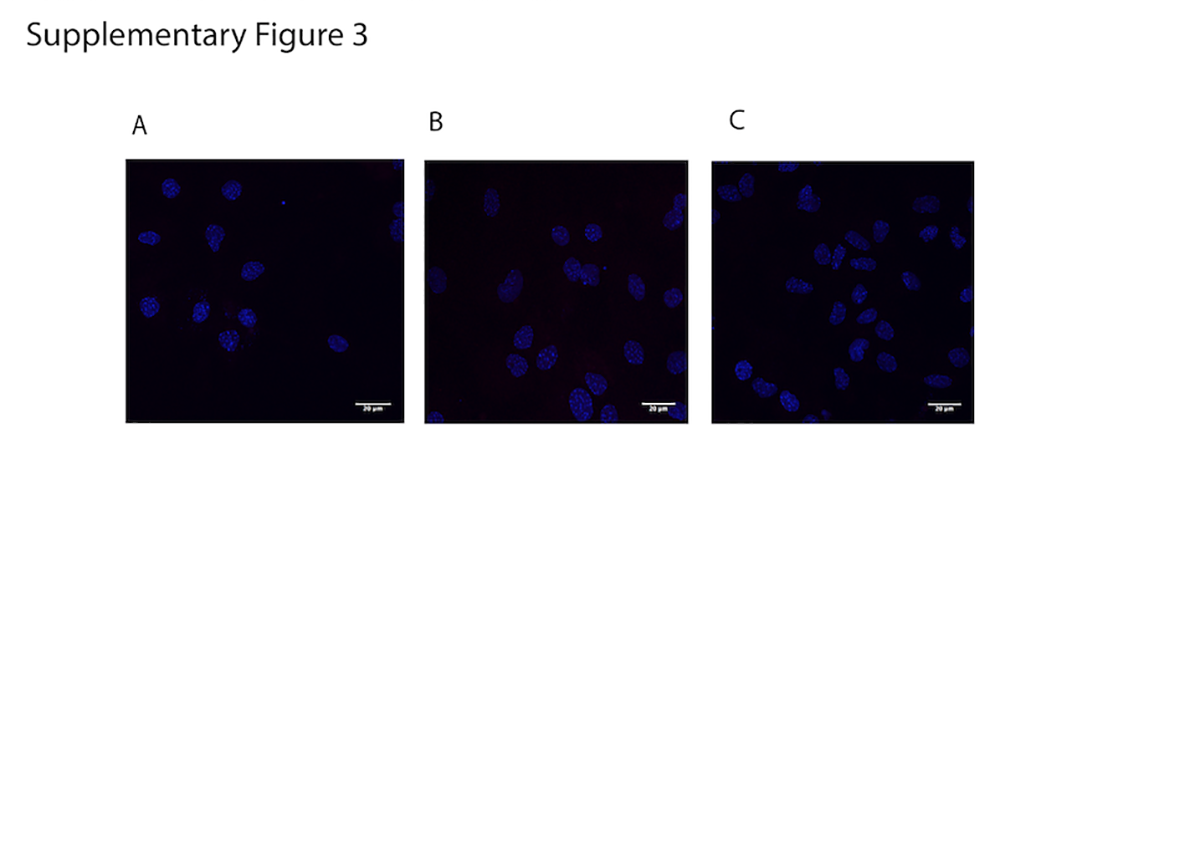

Supplement: Supplementary Figure 3 — Negative controls in PLA Assays. Negative controls for in situ PLA assays were performed in primary cultures of mice striatal neurons by omitting either the anti-CB1R (A), the anti-NCS1 (B) or the anti-calneuron-1 (C) antibodies. Scale bar: 30 μm. [file Image_3.TIFF]
